# Forty-One Plant Extracts Screened for Dual Antidiabetic and Antioxidant Functions: Evaluating the Types of Correlation between α-Amylase Inhibition and Free Radical Scavenging

**DOI:** 10.3390/molecules26020317

**Published:** 2021-01-09

**Authors:** Amir Bashkin, Manar Ghanim, Basheer Abu-Farich, Mahmoud Rayan, Reem Miari, Samer Srouji, Anwar Rayan, Mizied Falah

**Affiliations:** 1 Galilee Medical Center, Institute for Medical Research, Nahariya 2210001, Israel; AmirB@gmc.gov.il (A.B.); cute.mg.94@gmail.com (M.G.); reemmiari@yahoo.com (R.M.); dr.samersrouji@gmail.com (S.S.); 2 Faculty of Medicine in the Galilee, Bar-Ilan University, Safed 1311502, Israel; 3 Faculty of Science, Al-Qasemi Academic College, Baka EL-Garbiah 30100, Israel; af_basheer@qsm.ac.il (B.A.-F.); Mahmoud_ryan@hotmail.com (M.R.)

**Keywords:** hyperglycemia, blood glucose level, α-amylase, free radical scavenging, plant extract, antidiabetic, antioxidant

## Abstract

Dysregulation of glucose homeostasis followed by chronic hyperglycemia is a hallmark of diabetes mellitus (DM), a disease spreading as a worldwide pandemic for which there is no satisfactory dietary treatment or cure. The development of glucose-controlling drugs that can prevent complications of DM, such as hyperglycemia and oxidative stress, which contribute to the impairment of the key physiological processes in the body, is of grave importance. In pursuit of this goal, this study screened 41 plant extracts for their antidiabetic and antioxidant activities by employing assays to test for α-amylase inhibition and free radical scavenging activity (FRSA) and by measuring glucose uptake in L6-GLUT4myc cells. While extracts of *Rhus coriaria*, *Punica granatum, Olea europaea, Pelargonium* spp., *Stevia rebaudiana,* and *Petroselinum crispum* demonstrated significant α-amylase inhibition, the extracts of *Rhus coriaria* and *Pelargonium* spp. also demonstrated increased FRSA, and the extract of *Rhus coriaria* stimulated glucose uptake. These natural extracts, which are believed to have fewer side effects because they are prepared from edible plants, interfere with the process in the small intestine that breaks down dietary carbohydrates into monosaccharide and disaccharide derivatives, and thereby suppress increases in diet-induced blood glucose; hence, they may have clinical value for type 2 diabetes management. The *Pelargonium* spp. and *Rhus coriaria* extracts demonstrated the highest antidiabetic and antioxidant activities. Both plants may offer valuable medical benefits, especially because they can be taken as dietary supplements by patients with diabetes and can serve as sources of new, natural-based antidiabetic drug candidates. The enhancement of cellular glucose uptake stimulated by *Rhus coriaria* extract could lead to the development of clinical applications that regulate blood glucose levels from within the circulatory system. Isolating bioactive substances from these plant extracts and testing them in diabetic mice will significantly advance the development of natural drugs that have both antidiabetic and free radical-scavenging properties, likely with lesser side effects.

## 1. Introduction

Digestible starch, which is the principal source of carbohydrates and accounts for 40–60% of the energy intake in the human diet, gives rise to a prolonged release of glucose moieties in the lumen of the small intestine, which are subsequently absorbed into the bloodstream, causing increased postprandial glycemia [1,2]. As glucose makes up 80% of the total absorbable monosaccharides, high blood levels trigger cells in the pancreas to secrete insulin, a hormone that is important for regulating glucose homeostasis [3]. Insulin then signals adipose tissue to metabolize glucose into triglycerides, and skeletal muscle tissues metabolize it to generate energy; excess glucose is stored in the liver in the form of glycogen [4,5]. This tight regulatory process continuously maintains glucose homeostasis, even under prolonged fasting. Diabetes mellitus (DM) is a condition in which blood hyperglycemia occurs and cannot be controlled due to a lack of insulin activity [5,6,7]. DM is a metabolic disorder affecting hundreds of millions of people throughout the globe [8]; it is caused either by an insulin deficiency (type 1 diabetes) or by insulin resistance (type 2 diabetes) [9,10]. As 80% of the postprandial glucose is transported into muscles by the insulin-stimulated glucose transporter 4 (GLUT4) system, the resistance of muscle to insulin, which is typical in type 2 diabetes, disrupts glucose homeostasis in the whole human body [11,12,13]. Abnormalities in glucose homeostasis cause damage to tissues and organs and eventually lead to diabetic angiopathy, neuropathy, retinopathy, and nephropathy [14]. If this condition is not managed, these hyperglycemic abnormalities can also damage large blood vessels, which can progress to cardiovascular disease and stroke [15,16,17].

Because hyperglycemia can be managed to some extent by a low-carbohydrate diet, researchers and pharmaceutical companies seek methods to control it by modulating starch digestion [18,19]. However, although it can control blood glycemia, making starch resistant to digestion is not recommended due to safety concerns [18,19,20,21]. Inhibition of the activity of starch-digesting enzymes within the intestine, which delays carbohydrate digestion, reduces glucose absorption, and thereby decreases postprandial blood glucose levels, has become a widely accepted approach [22,23]. α-Amylase is a key player in dietary starch digestion, and because its inhibition plays an important role in reducing and regulating postprandial hyperglycemia [23,24,25,26], it is considered a desirable biological target for DM treatment [23]. The enzyme catalyzes carbohydrate digestion inside the small intestine by hydrolyzing 1, 4-glycosidic linkages and converting polysaccharides (starch) to disaccharides. The drug acarbose is a pseudotetrasaccharide containing a nonhydrolyzable, nitrogen-linked bond, which binds α-amylase and suppresses its activity through competitive, reversible inhibition. It is one of the second-line drugs in the treatment of diabetes after metformin [27,28]. However, its application is limited due to the cost of production and the side effects associated with its synthetic nature [29]. Therefore, in recent years, many studies have investigated the inhibition of α-amylase by natural products, such as plant extracts [30,31], and found that plants constitute a rich source for antidiabetic drug candidates acting via α-amylase inhibition [32,33]. Due to their natural characteristics and long process of evolutionary selection, plant extracts may induce fewer side effects when compared to synthetic drugs. In general, natural-based medicines have been proven safer [34,35,36] and are inherently better tolerated than synthetic drugs, and thus may be the best source of future medicines [37,38]. Specifically, many plants are used in the treatment of DM patients [39]; e.g., *Rhus coriaria*, a traditional Middle Eastern table spice, is recommended for the treatment of hyperlipidemia in diabetic patients [39,40].

In addition, there is a large body of evidence showing that oxidative stress is primarily induced by hyperglycemia and is associated with a key process in the initiation and progression of diabetic complications [41,42]; however, the precise mechanisms by which oxidative stress accelerates the development of diabetic complications are only partly known [43,44]. Inflammatory mediators are generated, and these are stimulated by hyperglycemia-induced oxidative stress, which leads to the production of free radical species [42,45]. Free radicals are an unstable species of molecule that pair up their odd free electrons by attacking healthy cells, causing loss of cell structure and/or function [46]. These damaged cells contribute greatly to degenerative diseases such as cancer, inflammation, immune system weakening, liver disease, brain dysfunction, cardiovascular conditions, diabetic renal failure, and others [47]. Plant antioxidants are free radical-scavenging agents that can control the detrimental effect of these unstable species on the human body [48] and may be useful in treating some diabetic complications. This work aimed to perform a large-scale screening of plant extracts that exhibit both inhibitory effects on α-amylase and free radical-scavenging properties with minimal side effects. To this end, 41 extracts from plants widely used by people in the Middle East as food and medication were screened for such activities. Once a plant extract that can act as both an antidiabetic and antioxidant is identified, it can be further studied to isolate its bioactive compounds.

## 2. Results

### 2.1. Screening Plant Extracts for Their Antidiabetic and Free Radical Scavenging Activities

Both the lack of effective drugs for the treatment of type 2 diabetes and the increasing prevalence of the disease have created an urgent need to improve drug therapies. Some studies have shown that natural, plant-derived products have safe characteristics, with minimal side effects, unlike many widely used synthetic drugs [37]. The use of natural drugs for the treatment of diabetes and its associated complications is expanding worldwide, and many plant-based products exhibit antidiabetic effects [37,49]. 

Our initial objective was to examine the antidiabetic and antioxidant effects of the extracts of 41 medicinal/edible local plants (Table 1). All methanolic extracts of these plants were screened for α-amylase inhibitory activity at a concentration of 2 mg/mL. Two of the extracts demonstrated high α-amylase inhibitory activity, suggesting antidiabetic activity; these were from *Pelargonium* spp. and *Rhus coriaria*, which exhibited 100 and 82% inhibition, respectively. We performed evaluations of the free radical scavenging activity (FRSA) of the extracts in parallel. Of the 41, *Cinnamomum aromaticum* extract showed the lowest EC_50_ for FRSA, and *Ceratonia siliqua* extract showed the highest (Table 1). Most importantly, *Pelargonium* spp. and *Rhus coriaria*, which had a strong inhibitory effect on α-amylase activity, also exhibited high levels of scavenging activity, indicating a dual function.

### 2.2. In Vitro α-Amylase Inhibition

Table 1 shows that *Pelargonium* spp. and *Rhus coriaria* display particularly high α-amylase inhibition and are as potent as acarbose (EC_50_ = 30 μg/mL [50]). Although the inhibition is clear, little is known about the mechanism by which it occurs. Further studies will shed light on the mechanism and help us to more effectively validate the use of the extract to treat type 2 diabetes. Furthermore, kinetic analyses are crucial to determining the type of inhibition exerted on α-amylase; the pattern of inhibition will suggest whether the active site of the enzyme is directly involved in the mechanism of action. In addition, the plant extract most likely contains some compounds that could serve as substrate analogs that compete for the active site of α-amylase. 

**Figure 1:**
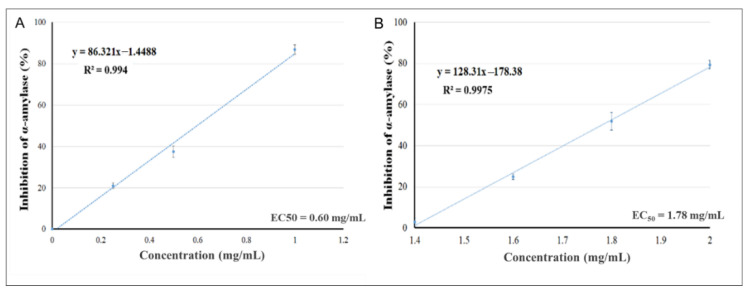
Inhibitory activity of *Pelargonium* spp. (**A**) and *Rhus coriaria* (**B**) against α-amylase.

The effects of increasing the concentrations of the plant extracts of *Pelargonium* spp. (Figure 1A) and *Rhus coriaria* (Figure 1B) to inhibit α-amylase activity were assessed, aiming to evaluate the values of EC_50_ (acarbose at concentration of 1.25 mM was used as a positive control). The EC_50_s of *Pelargonium* spp. and of *Rhus coriaria* were 0.60 and 1.78 mg/mL, respectively. The strong α-amylase inhibitory activity indicates the presence of active compounds that could inhibit the breakdown of complex carbohydrates into oligosaccharides, which could diminish the effects of carbohydrate consumption on postprandial hyperglycemia. 

An in vivo study to further confirm the results is required to validate and fractionate the active natural material to be developed for type 2 diabetes treatment.

*Pelargonium* spp. and *Rhus coriaria* may carry valuable medical benefits, especially because these substances may be used as nutritional supplements for people with diabetes.

### 2.3. Effects of the Plant Extracts on Free Radical Scavenging

The generation of reactive oxygen species (ROS) (or free radicals) plays an important role in the pathogenesis of diabetes, resulting in increased oxidative damage at the molecular and cellular levels. Excessive production of ROS in diabetic patients has been targeted with antioxidants in an effort to prevent and suppress the development of oxidative damage to DNA, proteins, and lipids within cells and tissues.

Plant products are the main source of natural antioxidant molecules that can eradicate or neutralize the damage caused by ROS [51]. They are currently available in many pharmaceutical venues and are used to manage diseases and health conditions, including to counteract ROS. Curcumin, a natural polyphenol derived from the rhizome of turmeric, and quercetin, an antioxidant derived from fruits and vegetables, have been shown to have potent FRSA and antidiabetic activity [52]. Polysaccharides from the leaves of Guava (GLP) [53], Lepidium Meyenii (MECA) [54], and Olive [55] have been evaluated for their antioxidant activity in vitro and have been found to display good FRSA.

The concentrations of the 41 plant extracts were increased, and the impacts on scavenging (antioxidative) activity were assessed using the in vitro 2,2-diphenyl-1-picrylhydrazyl (DPPH) assay. This is a reliable assay commonly used for the large-scale screening of plant extract samples for in vitro antioxidant activity. The results show that *Pelargonium* spp. and *Rhus coriaria* both have a low EC_50_ for FRSA (Table 2).

### 2.4. Correlation Analysis between α-Amylase Inhibition and Free Radical Scavenging Activity

Verifying the correlation between α-amylase inhibition and free radical scavenging activity will advance the subsequent screening of plant extracts by highlighting those with advantageous dual antidiabetic and antioxidant functions. The inhibition of α-amylase was first verified by screening all of the extracts at a concentration of 2 mg/mL (Table 1). The extracts that displayed a percentage of inhibition above 50% were then tested at lower concentrations to determine their IC_50_ values (Table 2). Two extracts were found to have IC_50_ values < 2 mg/mL—*Pelargonium* spp. and *Rhus coriaria.* Their IC_50_ values, as measured by dose-response experiments, were 0.60 and 1.78 mg/mL, respectively. Treatment with 2 mg/mL of *Olea europaea, Petroselinum crispum, Stevia rebaudiana*, and *Punica granatum* inhibited α-amylase by 42.6, 37.5, 35.7 and 27.4%, respectively (Table 1).

**Figure 2:**
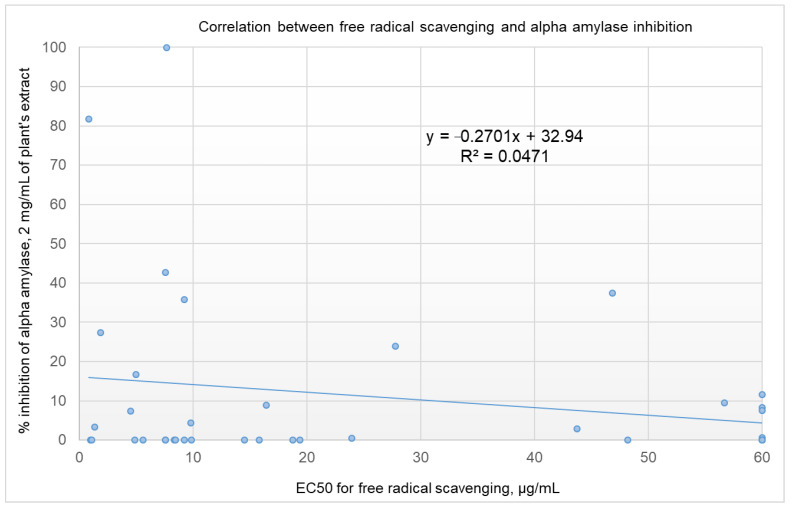
Correlation between the percentage of inhibition of α-amylase by 2 mg/mL of plant extract and the EC_50_ for FRSA. A free radical scavenging EC_50_ > 60 µg/mL is set at 60 µg/mL.

**Figure 3:**
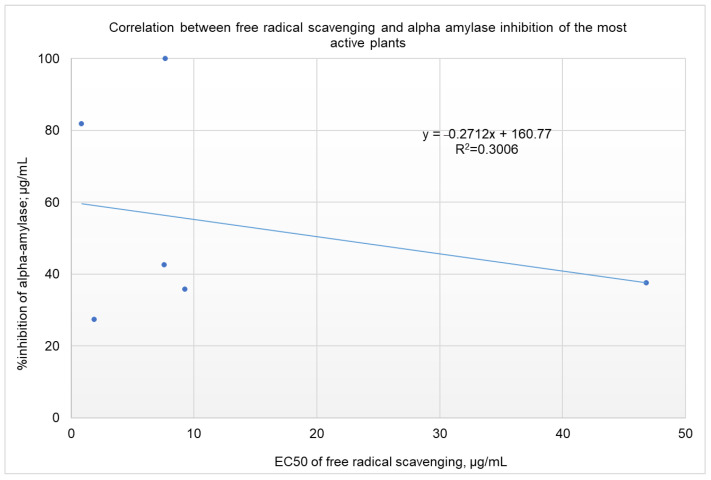
Correlation between the percentage of inhibition of α-amylase and the EC_50_ for FRSA for the six most active plant extracts.

Rules-based analysis using Matthews correlation coefficient (MCC) scores and enrichment factors as criteria for the evaluation of the efficiency of the model revealed that plant extracts with a free radical-scavenging EC_50_ ≤ 10 µg/ml showed somewhat better inhibition of α-amylase (see Table 3). The values for the enrichment factor, the MCC, accuracy, and precision were 1.7, 0.286, 0.61, and 0.25, respectively. These findings are of great importance for screening projects. Identifying extracts with a 1.7 order of enrichment in inhibiting α-amylase from among plant extracts with an EC_50_ of FRSA ≤ 10 µg/mL could save time and money in high-throughput screening. It is worth noting that, taking these results together, we might be deceived and conclude that there is no correlation between the inhibition of α-amylase and the free radical scavenging activities of the screened extracts (as shown in Figure 2, *R^2^* = 0.0471). For the six most active plant extracts, there was a low correlation, with *R^2^* = 0.3006 (shown in Figure 3). Five of the six active plant extracts (i.e., *Pelargonium* spp., *Punica granatum*, *Olea europaea, Rhus coriaria*, and *Stevia rebaudiana*) showed relatively high FRSA, while the activity for *Petroselinum crispum* was relatively low.

**Figure 4:**
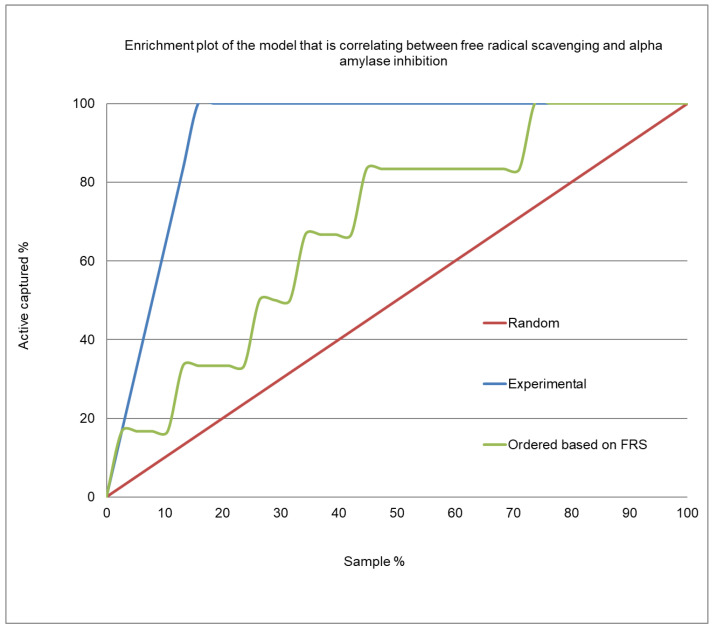
Enrichment plot of the prediction model for α-amylase inhibition by the plant extracts, based on their FRSA.

**Figure 5:**
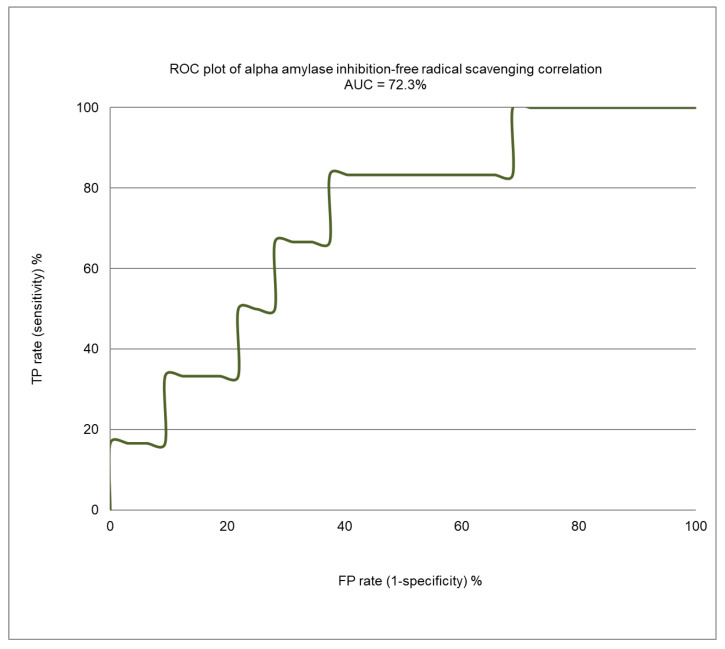
A receiver operating characteristic (ROC) curve showing the performance of the FRSA-α-amylase inhibition correlation model.

Figure 4 presents the enrichment plot, and Figure 5 shows the receiver operating characteristic (ROC) plot for the α-amylase inhibition/free radical-scavenging correlation model. The area under the curve (AUC) that was obtained for the current model was 0.723 (Figure 5), indicating a very weak correlation between α-amylase inhibition and FRSA. The enrichment plot (Figure 4) illustrates how quickly the active extracts of plants can be identified when they are sorted according to their FRSA. In-depth analysis reveals that the shape seen in Figure 4 fits well with the conclusions drawn from the detailed analysis of Table 3, which shows that the plant extracts with an EC_50_ for FRSA ≤ 10 µg/mL exhibit strong α-amylase inhibition of a 1.7 order of magnitude.

In Table 3, the Matthews correlation coefficient (MCC) scores and enrichment factors are utilized as criteria in the evaluation of the models. The calculations were based on the assumption that a 250 µg/mL-plant extract that produced α-amylase inhibition ≥25% was active (a true positive); extracts with inhibition below 25% were considered inactive. Six out of the 41 tested plant extracts showed an inhibition of α-amylase ≥25%.

Figure 4 shows the enrichment plot, and Figure 5 shows the ROC plot, of the FRSA-α-amylase inhibition correlation model. The enrichment plot illustrates how quickly the active extracts of plants can be identified when they are sorted according to their FRSA. In the enrichment plot, a close-to-perfect curve reflects the high prioritization power of the proposed model.

According to the results of the above analysis, there is, to some extent, a correlation between α-amylase inhibition and the FRSA of the tested plant extracts. Plant extracts with FRSA EC_50_ ≤ 10 μg/mL have a higher chance (a 1.7 order of magnitude) of inhibiting α-amylase enzyme than plant extracts with FRSA EC_50_ > 10 μg/mL. This piece of information is significant because it helps to reduce the number of plant extracts that have to be included in subsequent investigational screenings and highlights natural products that have dual antidiabetic and antioxidant properties.

### 2.5. Effects of the Plant Extracts on Glucose Uptake in Skeletal Muscles

*Pelargonium* spp. and *Rhus coriaria* extracts were further investigated for their capacity to stimulate glucose uptake by the L6-GLUT4myc skeletal muscle cell line. First, the concentration ranges of the substances at which the muscle cells preserve their viability were determined. 

#### 2.5.1. Effects of the Plant Extracts on Cell Viability

**Figure 6:**
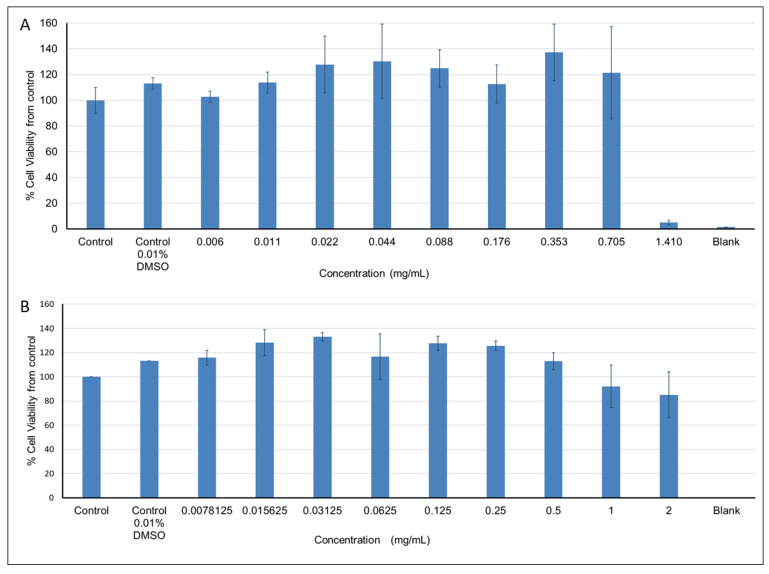
The effects of increasing concentrations of the plant extracts on the viability of L6-GLUT4myc cells: (**A**) *Pelargonium* spp. and (**B**) *Rhus coriaria* extracts.

*Pelargonium* spp. at concentrations ranging between 0.006 and 0.705 mg/mL had no effect on cell viability, while higher concentrations resulted in 85–95% cell viability (Figure 6A). Exposure of cells to *Rhus coriaria* extract at concentrations between 0.0078125 and 0.5 mg/mL had no effect on cell viability, while higher concentrations resulted in decreased cell viability (up to 15%) compared to the control (Figure 6B). Viability above 100% (the control), observed at some extract concentrations, can be explained by the absorption of some components within the extracts that had not been washed out.

#### 2.5.2. Effects of *Pelargonium* spp. and *Rhus coriaria* Extracts on GLUT4 Translocation

**Figure 7:**
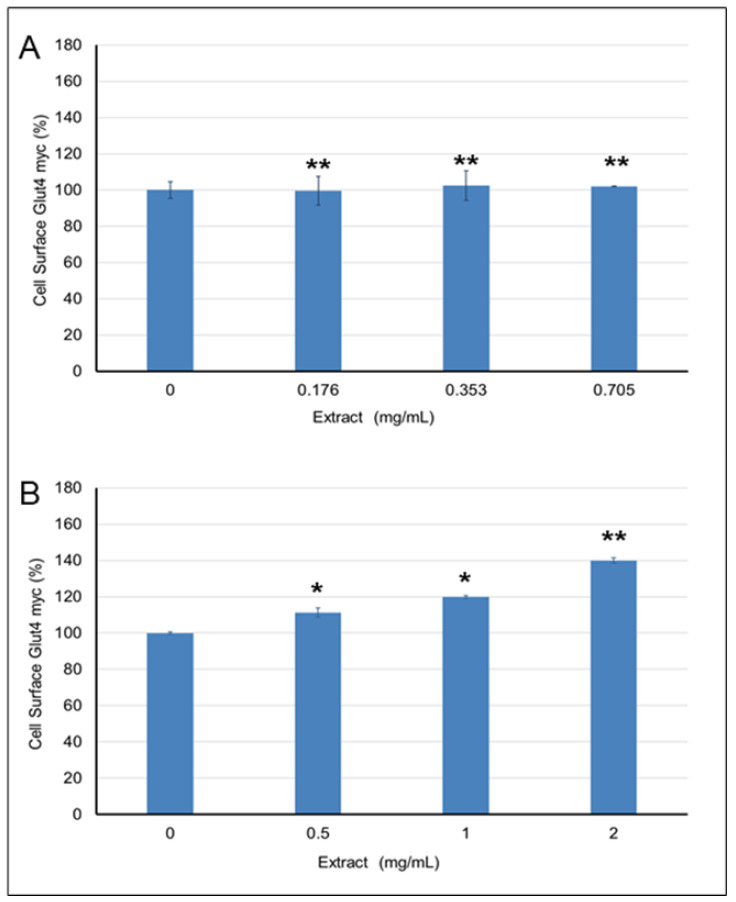
The effect of: (**A**) *Pelargonium* spp. and (**B**) *Rhus coriaria* extracts on GLUT4 translocation. The results represent a mean ± standard deviation of three experiments (*n* = 3). One asterisk (*) indicates *p* < 0.05. Two asterisks (**) indicate *p* < 0.001.

The effects of *Pelargonium* spp. and *Rhus coriaria* extracts on GLUT4 translocation in L6-GLUT4myc cells were assessed by exposing cells to the extracts at three concentrations that were found to inhibit α-amylase and show FRSA. The solvent in which the extract was dissolved was used as a negative control. Insulin was used as a positive control, and solvent without extract was used as a negative control. No effect of the *Pelargonium* spp. extract on GLUT4 translocation was observed (Figure 7A). In contrast, treatment of cells with 0.5, 1, or 2 mg/mL of *Rhus coriaria* extract increased the translocation of GLUT4 by 111% (1.11-fold, *p* < 0.05), 119% (1.19-fold, *p* < 0.05), and 139% (1.39-fold, *p* < 0.01), respectively, as opposed to the control (with 0% concentration of the extract).

These results are consistent with those of studies reporting increased glucose uptake as a result of enhanced GLUT4 levels in L6 cells [56,57,58]. Previous studies have also showed decreased GLUT4 expression in skeletal muscle and adipose tissue in GLUT4 +/− heterozygous mice and, as a result, a decrease in glucose uptake in muscle cells and an increase in peripheral insulin resistance, followed by an increase in blood glucose levels [59,60]. However, when the transgenic mice overexpressed GLUT4, increased sensitivity to insulin was observed [61].

Therefore, deficiencies in glucose uptake in skeletal muscle cells are mostly linked to defects in the insulin signaling pathway and are probably caused by changes in GLUT4 transporter translocation to the cell surface [62]. Therefore, extracts such as *Rhus coriaria* (Figure 7B), which mobilize normal GLUT4 transporter levels to the cell surface, may be useful in the development of drugs for treating type 2 diabetes. *Rhus coriaria* extract also exhibited increased anti-α-amylase, as well as FRSA. This higher activity might be due to the presence of supportive phytochemicals that need to be isolated and chemically identified, after which the biological activity of the individual phytochemicals, along with the mechanisms involved, should be verified. 

## 3. Materials and Methods 

### 3.1. Materials

All of the plants used in this study were purchased from Al Alim Ltd. (Medicinal Herb Center, Zippori, Israel). DPPH and the solvents were purchased from Sigma, Israel. Acarbose, starch, glucose, and α-amylase were all purchased from Sigma-Aldrich, Saint Louis, MO, USA.

### 3.2. Preparation of Plant Extracts 

To produce each extract sample, approximately 1 g of dried plant material was packed in a tube and soaked in methanol (10 mL for each gram of material), sonicated for 75 min at 40 °C, then left for 3 h to cool for complete extraction. The methanolic extracts were then filtered through grade-1 Whatman paper, and the filtered solution was concentrated by evaporation to dryness in a vacuum, dissolved in DMSO to a concentration of 100 mg/mL, and stored at 4 °C for future activity testing. 

### 3.3. α-Amylase Activity

α-Amylase inhibitory activity was tested using a standard assay, with minor modifications [13]. Reaction mixtures containing 20 μL of extracts at different concentrations (8, 4, 2, 1, 0.5 mg/mL), 50 μL phosphate buffer (100 mM, pH = 6.8), and 10 μL of α-amylase (2 U/mL) were incubated for 5 min at 37 °C, in 96-well plates. Thereafter, 20 μL of 1% soluble starch (100 mM phosphate buffer, pH 6.8) was added, and the mixture was incubated for 5 min, at 37 °C; the buffer did not include magnesium or calcium and served as the substrate for the reaction. Afterward, 100 μL of DNS color reagent was added, and the mixture was boiled for 10 min, after which the absorbance was measured at 540 nm with a microplate reader. Acarbose was used as a positive control, and each experiment was carried out in quadruplicate. The percentage of α-amylase inhibition was calculated according to Equation (1):

(1) $$\mathrm{Inhibitory}\;\mathrm{activity}\left(\%\right)=\frac{\mathrm{absorbance}\;\mathrm{in}\;\mathrm{the}\;\mathrm{presence}\;\mathrm{of}\;\mathrm{test}\;\mathrm{substance}}{\mathrm{absorbance}\;\mathrm{of}\;\mathrm{control}}\times 100$$

### 3.4. Free Radical Scavenging Activity (FRSA)

The FRSA of the methanolic extracts of the various plants was measured using the DPPH assay protocol, with minor modifications [14]. The DPPH was serially diluted in pasteurized water, and the tests were carried out in 96-well cell culture plates. Diluted DPPH solution (200 ppm, 100 μL) was added to 100 μL of the methanolic plant extracts. The mixture was shaken and allowed to stand for 30 min in the dark at room temperature. Then the absorbance of the solution was measured at 517 nm and converted into a percentage of FRSA, using Equation (2):

(2) FRS(%)={1−[(Asample−Ablank1)(Acontrol−Ablank2)]}×100,

where *A_sample_* is the absorbance of the plant extract and the DPPH mixture, *A_blank1_* is the absorbance of the plant extract, *A_control_* is the absorbance of the ethanolic solution of DPPH, and *A_blank2_* is the absorbance of ethanol.

FRSA is expressed in terms of the half-maximal effective concentration (EC_50_), i.e., the amount of antioxidants necessary to decrease the initial DPPH absorbance by 50%. The EC_50_ value for each plant extract was determined by calculating the value using the equation of the linear part of the graph. Gallic acid (100 µg/mL) was used as a positive control.

### 3.5. Cell Viability

The viability of cells exposed to different concentrations of the plant extracts was assessed using the Cell Proliferation Kit (XTT based) (Biological Industries, Beit Haemek, Israel). The assay is based on the ability of the metabolically active cells to reduce the amount of applied dye, whose color changes to orange, the intensity of which is proportionate to the number of cells. The intensity of the color was measured with a spectrophotometer at a wavelength of 450 nm.

L6-GLUT4myc cells (2 × 10^4^/well) were seeded in 100 μL of growth medium in 96-well plates and incubated for 24 h. The *Pelargonium* spp. and *Rhus coriaria* extracts were then added at various concentrations, and the plates were incubated for 20 h at 37 °C. The next day, XTT reagents were added to the plate (medium was added to the control wells) and incubated for 2 h at 37 °C. The control samples were treated with 0.01% DMSO (the solvent in which the plant extracts were dissolved). Cell viability was evaluated according to the manufacturer’s instructions, using a Thermo Scientific Varioskan LUX Multimode Microplate Reader at 450 nm. The measured absorbance was subtracted from the reference absorbance (620 nm), and each experiment was repeated independently three times. 

### 3.6. Glucose Uptake

Glucose uptake into skeletal muscle is mostly performed via GLUT4, which is recruited from the cytosol to the cell surface, a process that is stimulated by insulin or other antidiabetic molecules [11,12,13,63]. GLUT4 content on cell surfaces has been documented to be positively correlated with glucose uptake activity in skeletal muscle cells [63]. L6-GLUT4myc cells are the only cellular model available for investigating glucose uptake and GLUT4 translocation without requiring cell permeabilization or fractionation. Cell-surface GLUT4-myc levels were quantitated using a previously published protocol [63]. In brief, rat L6 muscle cell-line cells stably expressing myc-tagged GLUT4 were cultured (5% CO_2_, 37 °C) in a complete growth medium composed of α-MEM, 10% FBS, 100 U/mL penicillin, and 0.1 mg/mL streptomycin. The cells (2 × 10^5^/well) were seeded onto 24-well plates and cultured for 24 h, then treated with natural products and plant extracts for 20 h. Next, they were incubated with serum-free medium for 3 h and then treated with 1 µM of insulin (as a positive control) or with plant extracts at 37 °C for 20 min. The cells were then washed twice with ice-cold PBS, fixed with 3% paraformaldehyde (PFA) (10 min), incubated with 0.1 M glycine, blocked with 3% (*v*/*v*) goat serum for 30 min, and reacted with polyclonal anti-myc antibody for 1 h at 4 °C. After that, the cells were washed 5 times with wash buffer and reacted with anti-rabbit IgG for 1 h at 4 °C, then washed another 5 times with wash buffer. Following this, 0.2 mL of 3,3′,5,5′-tetramethylbenzidine (TMB) was added to each well, and the cells were incubated in the dark at RT for 5–10 min, after which 3N hydrochloric acid (HCl) (0.2 mL/well) was added to terminate the reaction. Finally, the absorbance of the supernatants was measured using a Thermo Scientific Varioskan LUX Multimode Microplate Reader at 492 nm.

### 3.7. Model Assessments

Parameters, such as the Matthews correlation coefficient (MCC), accuracy, the precision enrichment factor, and the area under the ROC curve (AUC), were calculated to assess the quality of the free radical scavenging α-amylase activity correlation models (see Equations (3)–(6)).

Equation (3). Matthews correlation coefficient (MCC)

(3) $$MCC=\frac{\left(PN)-(P_{f}N_{f}\right)}{\sqrt{\left(N+N_{f})(N+P_{f})(P+N_{f})(P+P_{f}\right)}},$$

where *P*, *N*, *P_f_*, and *N_f_* are the number of true positive, true negative, false positive, and false-negative predictions, respectively. A perfect prediction yields *MCC* = 1.0, while a random performance yields *MCC* = 0.0 and *MCC* = −1.0 indicates a completely erroneous prediction.

Equation (4). Accuracy

Accuracy = (*P* + *N*)/(*P* + *N* + *P_f_* + *N_f_*) (4)

Equation (5). Precision

Precision = *P*/(*P* + *P_f_*) (5)

Equation (6). Enrichment factor

*EF* = *T_FRS_*/*T_RS_*, (6)

where *T_FRS_* is the percentage of actives when using the FRS threshold criterion, and *T_RS_* is the percentage of actives by random selection.

### 3.8. Statistical Analyses

Statistical analyses were performed using SPSS software, and means were compared using the two-tailed parametric test (Student’s *t*-test). Statistical significance levels were set at * *p* < 0.05, ** *p* < 0.01, and *** *p* < 0.001.

## 4. Conclusions

Since ROS are abundant and massively produced in diabetic patients, we aimed to draw a correlation between the inhibition of α-amylase and the FRSA of plant extracts, with the ultimate goals of identifying plant extracts possessing both antioxidant and antidiabetic activity and facilitating the screening process for identifying antidiabetic natural products, preferably those exhibiting both antidiabetic and antioxidant activity. To this end, the FRSA of several plant extracts was assessed using the DPPH assay, while α-amylase inhibition was measured using the DNS assay. *Pelargonium spp., Punica granatum*, *Olea europaea, Rhus coriaria*, *Stevia rebaudiana,* and *Petroselinum crispum* plant extracts were found to display some anti-α-amylase activity. An in-depth analysis of possible correlations between FRSA and α-amylase inhibition revealed that the plant extracts which exhibited an FRSA EC_50_ ≤ 10 μg/mL showed some degree of enrichment for anti-α-amylase activity (at a 1.7 order of magnitude). These findings are of great importance as they may significantly shorten screening processes, saving both time and money.

The *Rhus coriaria* extract exhibited α-amylase inhibition, a high level of free radical scavenging, and increased GLUT4 translocation onto cell membranes in a dose-dependent manner. Hence, the extract of *Rhus coriaria* may be useful as a nutraceutical or as a source of drugs to treat type 2 diabetes disease. Its high activity might be due to the presence of supportive phytochemicals, which will need to be isolated and chemically identified, after which the biological activity of the individual phytochemicals, along with their mechanisms, should be verified. 

Due to these considerations, our next study will use a novel in-house bioassay-guided fractionation approach [64,65,66,67] and in silico-based modeling techniques [68,69,70,71,72] to isolate substances from bioactive plant extracts that exhibit antidiabetic activity. We suggest that subsequent studies validate the conclusions reached in the current study by testing other antidiabetic parameters. In addition, the biological activity of the isolated substances should be tested in diabetic mice. Preventing oxidative stress and balancing blood glucose levels in diabetic mice by orally administered natural products will significantly advance the development of natural drugs exhibiting both antidiabetic and antioxidant functions.

## Figures and Tables

**Table 1:** Medicinal/edible plant extract-driven inhibition of α-amylase activity and the EC_50_ values for free radical-scavenging activity. Active extracts that inhibit α-amylase in a ratio above 25% are labeled in bold.

No.	Plant Name	% Inhibition of α-Amylase Activity	Free radical Scavenging EC_50_ (μg/mL)
1	*Rhus coriaria*	**81.75 ± 0.51**	0.87
2	*Curcuma longa*	0	0.98
3	*Green tea*	0	1.10
4	*Camelia sinensis*	3.27 ± 2.24	1.38
5	*Punica granatum*	**27.39 ± 1.30**	1.89
6	*Angelica sylvestris*	7.41 ± 1.41	4.53
7	*Thymus vulgaris*	0	4.87
8	*Mentha piperita*	16.67 ± 0.89	4.98
9	*Humulus lupupus*	0	5.63
10	*Olea europaea*	**42.62 ± 1.17**	7.59
11	*Thymus capitatus*	0	7.59
12	*Vitis vinifera*	0	7.59
13	*Pelargonium* spp.	**99.89 ± 0.68**	7.69
14	*Rubus idaeus*	0	8.33
15	*Lippia citriodora*	0	8.46
16	*Corchorus olitorius*	0	9.22
17	*Stevia rebaudiana*	**35.73 ± 1.25**	9.26
18	*Rosmarinus officinalis*	4.37 ± 1.04	9.80
19	*Laurus nobilis*	0	9.88
20	*Cymbopogon citratus*	0	14.53
21	*Salvia officinalis*	0	15.82
22	*Ocimum basilicum*	8.90 ± 2.33	16.46
23	*Cynara cardunculus*	0	18.78
24	*Senna acutifolia*	0	19.40
25	*Melissa officinalis*	0.51 ± 0.93	23.96
26	*Avena sativa*	23.85 ± 0.72	27.78
27	*Cuminum cyminum*	2.86 ± 1.84	43.73
28	*Petroselinum crispum*	**37.48 ± 0.33**	46.82
29	*Chamomile*	0	48.19
30	*Ceratonia siliqua*	9.43 ± 0.87	56.66
31	*Centaurea*	11.63 ± 0.86	>60
32	*Gentiana*	8.33 ± 1.14	>60
33	*Gundelia tournefortii*	0.62 ± 1.24	>60
34	*Malva*	7.53 ± 1.02	>60
35	*Petroselinum*	0	>60
36	*Portulaca oleracea*	0	>60
37	*Vitex agnus-castus*	0	>60
38	*Zingiber officinale*	0	>60
39	*Foeniculurn vulgare*	0	n.d.
40	*Urtica urens/pilulifera*	0	n.d.
41	*Cinnamomum aromaticum*	- *	0.60
	*Acarbose (1.25 mM)*	**89.67 ± 4.8**	n.d.

* *Cinnamomum aromaticum* extract was insoluble; therefore, it was excluded from the enzymatic assay. n.d. means not determined.

**Table 2:** The inhibition of α-amylase and free radical scavenging by *Pelargonium* spp. and *Rhus coriaria,* the two most effective plant extracts with dual functionality.

Plant Name	Plant Picture	EC_50_ of α-Amylase Inhibition	EC_50_ of Free Radical Scavenging
*Pelargonium* spp.	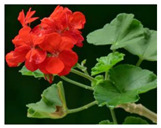	0.60 mg/mL	7.69 µg/mL
*Rhus coriaria*	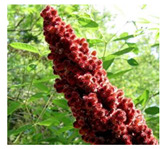	1.78 mg/mL	0.87 µg/mL

**Table 3:** EC_50_ cutoffs for free radical scavenging activity (FRSA).

FRSA EC_50_ Cutoffs				All
	≤5 µg/mL	≤10 µg/mL	≤60 µg/mL	-
Number of active plants (true positives) ^1^	2	5	6	6
Number of inactive plants (false positives) ^2^	7	15	25	35
Number of inactive plants (true negatives) ^3^	28	20	10	0
Number of active plants (false negatives) ^4^	4	1	0	0
Precision	0.22	0.25	0.194	0.146
Accuracy	0.73	0.61	0.39	0.146
Enrichment factor	1.5	1.7	1.3	1.0
MCC	0.114	0.286	0.235	0.0

^1^ Number of plant extracts (250 µg/mL) with an FRSA EC_50_ less than the certain threshold and inhibition of α-amylase ≥ 25%. ^2^ Number of plant extracts (250 µg/mL) with an FRSA EC_50_ lower than the indicated threshold and inhibition of α-amylase < 25%. ^3^ Number of plant extracts (250 µg/mL) with an FRSA EC_50_ higher than the indicated threshold and inhibition of α-amylase < 25%. ^4^ Number of plant extracts (250 µg/mL) with an FRSA EC_50_ higher than the indicated threshold and inhibition of α-amylase ≥ 25%.

## Data Availability

Data is contained within the article.

## Sample Availability

Samples of the compounds/plants are available from the corresponding authors.
